# Benzoic Acid and the Benzoates as Urinary Antiseptics

**Published:** 1911-03-11

**Authors:** L. C. V. Hardwicke


### The General Practitioner's Column.

[Contributions to this Column are invited, and if accepted, will be paid for.]

### BENZOIC ACID AND THE BENZOATES AS URINARY ANTISEPTICS.

By L. C. V. HARDWICKE, M.B., B.Ch.

In these days of specialised therapeutics and proprietary drugs we are apt to forget some of the older and, in many instances, more efficacious remedies. Clinical experience is overwhelming in favour of the benzoates in septic conditions of the urinary passages, especially in chronic cystitis; this fact is—or ought to be—well known, but how often are they given? On referring to "The Pharmacopœias of the London Hospitals" (Squire, eighth edition, 1910) I find that Guy's and King's are the only two hospitals where there are recognised prescriptions containing benzoates used as urinary antiseptics. The more usual method is to prescribe urotropin—a drug from the use of which I have seen very little, if any, benefit, and do not now prescribe—and to irrigate the bladder with various antiseptic solutions such as boracic acid, silver nitrate or argyrol, perchloride of mercury, etc., an unpleasant and usually painful method which is practically impossible to be carried out thoroughly by a busy general practitioner, especially in a district where hospital treatment is not available; and one that takes time, and in which the action of the antiseptic is not lasting, and in some cases even does harm, either by the introduction of fresh organisms, as when boracic acid is used, or by irritating the mucous membrane of the bladder and urethra, as when a more powerful antiseptic is used.

"The British Pharmaceutical Codex," 1907, says: "Benzoic acid combines with glycocol in its course through the kidneys and is excreted in the urine as hippuric acid. It is used in cystitis and other genito-urinary diseases to diminish the alkalinity and putridity of the urine." The benzoates act in the same way: "Sodium benzoate has the same action as benzoic acid, but it is less irritating and is also preferred on account of its ready solubility in water." And again: "Ammonium benzoate has an action identical with that of sodium benzoate, except that it is more rapidly absorbed." Clinically I have almost invariably found that after having been administered for a few days in fairly large doses the benzoates not only "diminish the alkalinity and putridity of the urine," but render it acid; and if we take this in conjunction with the fact that there are practically only three microbes of importance—namely, bacillus typhosus, tubercle bacillus, and bacillus coli communis—that can thrive in acid urine, it is seen that in the benzoates we have a powerful remedy in combating cystitis and septic conditions of the genito-urinary passages.

The benzoates are invaluable: (1) in chronic or sub-acute cystitis, especially where the urine is strongly alkaline and contains much pus; (2) as a preventive against infection in gradual dilatation of a stricture with bougies; (3) when a catheter has to be passed frequently, as in enlarged prostate; and (4) after perineal section for stricture by keeping the wound clean and stimulating healing. I usually prescribe the ammonium salt; it is soluble in 1 in 6 of water and is readily absorbed. The B.P. dose is 5 to 15 grains; but this is too small. I give 20 or 25 grains, four or even six times a day, and have never seen any ill-effects from these larger doses, but, on the contrary, the general health of the patients has improved, especially old people with chronic bronchitis with scanty and tenacious expectoration. I have also seen great benefit follow its use in chronic nervous diseases with vesical crises, the bladder symptoms becoming greatly diminished or altogether disappearing after a few weeks' treatment.

Case I.—C. H. R., forty-nine, first seen on January 4. A stricture just in front of anterior layer of triangular ligament; urine contained much pus, and was strongly alkaline.

℞ Ammon. benzoat. ... ... gr. xx.

Tinct. hyoscyami ... ... m xx.

Infusi buchu ... ... ad. ʒj.

Four times daily.

The amount of pus gradually lessened until on January 11 there was none and the urine was acid. On January 14 I commenced dilatation with bougies, passing a larger size every alternate day, until on January 27 the urethra was well dilated. The mixture was continued during this time, the urine remaining acid and free from pus.

Case II.—T. W., fifty-five, first seen on March 11. Slight pain at end of micturition; the urine was alkaline and contained pus. For some two and a half years a catheter had been passed about once a month for spasmodic stricture, the last time eleven days previously.

℞ Ammon. benzoat. ... ... gr. xx.

Aq. chloroformi ... ad ʒj.

Four times daily.

The urine was examined daily; on the fifth day it was acid and contained no pus, and on the seventh day there was no pain on or after micturition.

Case III.—C. T., sixty-six, first seen on March 18. Enlarged prostate, and catheters had been passed at intervals for three or four months. Urine was alkaline and contained a large quantity of pus.

℞ Ammon. benzoat. ... ... gr. xx.
Tinct. nuc. vom. ... ... m xx.
Aq. chloroformi ... ad ʒj.
Four times daily.

In five days the urine was slightly acid, the pus being greatly diminished in quantity. Subsequently the urine contained no pus and was distinctly acid.

Case IV.—W. H. M., fifty-eight, first seen on May 12. Two operations for stricture at St. Peter's Hospital eighteen years ago, and since has been in the habit of passing a catheter on himself. No other illnesses. The patient came to me from another practitioner, who wrote: "W. H. M. has been in the habit of passing a catheter on himself at intervals for some years. For four months now he has had cystitis. He has been treated with urotropin, gr. x. t.d., and bladder washed out with various antiseptics. The urine for a time improved, but still contains pus. He is now having his bladder washed out daily, a No. 7 silver catheter being used, with 1 in 20,000 hydrarg. perchlor., and taking mist. copaibæ (m xv.) t.d." The patient had a yellowish "toxic" appearance and looked ill. Urine was passed quite freely and without pain; it was alkaline and contained a considerable amount of pus. He was given the same prescription as Case III. Two days later, May 14, the urine was slightly acid, but still contained pus; on May 15 the pus was much less, and on May 17 the urine was strongly acid and contained no pus. I saw him up to June 9, when treatment ceased; he then looked well; the urine remained strongly acid and free from pus. Beyond having the mixture and being on a restricted light fluid diet the first five days—*i.e.* until the urine was free from pus—no other treatment was carried out.

